# Abstract Spatial Concept Priming Dynamically Influences Real-World Actions

**DOI:** 10.3389/fpsyg.2012.00361

**Published:** 2012-09-27

**Authors:** Sarah M. Tower-Richardi, Tad T. Brunyé, Stephanie A. Gagnon, Caroline R. Mahoney, Holly A. Taylor

**Affiliations:** ^1^Cognitive Science Team, U.S. Army NSRDECNatick, MA, USA; ^2^Department of Psychology, Tufts UniversityMedford, MA, USA; ^3^Department of Psychology, Stanford UniversityStanford, CA, USA

**Keywords:** spatial cognition, embodied cognition, masked priming, mouse tracking, abstract concepts

## Abstract

Experienced regularities in our perceptions and actions play important roles in grounding abstract concepts such as social status, time, and emotion. Might we similarly ground abstract *spatial* concepts in more experienced-based domains? The present experiment explores this possibility by implicitly priming abstract spatial terms (north, south, east, west) and then measuring participants’ hand movement trajectories while they respond to a body-referenced spatial target (up, down, left, right) in a verbal (Exp. 1) or spatial (Exp. 2) format. Results from two experiments demonstrate temporally dynamic and prime biased movement trajectories when the primes are incongruent with the targets (e.g., north – left, west – up). That is, priming abstract coordinate directions influences subsequent actions in response to concrete target directions. These findings provide the first evidence that abstract concepts of world-centered coordinate axes are implicitly understood in the context of concrete body-referenced axes; critically, this abstract-concrete relationship manifests in motor movements, and may have implications for spatial memory organization.

## Introduction

Spatial thinking is a fundamental part of our daily routines; we rely on spatial memory to navigate around our homes and to work, and we constantly acquire novel spatial experiences as we move through our environment. Traditional theories largely assume that our mental representations of space are tightly bound to specific experiences. For instance we can mentally represent ground-level views after navigating on foot, and we remember environmental structure after viewing a map (Tolman, 1948; Mou and McNamara, 2002; Jeffery and Burgess, 2006). More recent work, however, has demonstrated that memories of environments are flexible, malleable, and highly vulnerable to factors both internal (e.g., experience level, goals, handedness, spatial skills, preferences, and heuristics) and external (e.g., environment complexity and density) to an individual (i.e., Taylor et al., 1999; Waller, 2000; Hegarty et al., 2006; Brunyé and Taylor, 2009; Gyselinck et al., 2009; Brunyé et al., 2010a, 2012b). In this context, memories for environments experienced from a first-person (i.e., *egocentric*) perspective may also integrate *allocentric* spatial knowledge that is more abstract in nature, relying on fixed, self-removed coordinate terms.

It is unclear how exactly people might represent such abstract world-centered reference systems in spatial memory. A growing body of research suggests that understanding abstract concepts involves making connections to experience-based domains (Boroditsky and Prinz, 2008). For instance, people rely upon the concrete horizontal spatial axis when thinking about time (Boroditsky and Ramscar, 2002), the vertical spatial axis when thinking about social status (Piaget, 1927/1969; Tversky et al., 1991; Casasanto and Lozano, 2006; Gagnon et al., 2011), and both horizontal and vertical spatial axes when thinking about affective valence (Meier and Robinson, 2004; Casasanto and Chrysikou, 2011). In each of these cases, people appear to use concrete perceptual, sensory, and interoceptive experiences with the world to structure abstract thought.

The notion that people integrate bodily experiences into mental representations is foundational to theories of embodied cognition (Lakoff and Johnson, 1999; Barsalou, 2008). Strong behavioral and neural evidence exists in favor of these theories; for instance, reading about an object primes the actions typically performed upon the object (Borghi et al., 2004), and reading action-related words biases subsequent hand movements (Glenberg and Kaschak, 2002; Richardson et al., 2003) and spontaneously activates motorically relevant areas of the pre-motor cortex (Kemmerer et al., 2008). Recent work further suggests that processing abstract language engages perceptual and action-based representations; for instance, abstract indications of movement direction (e.g., *delegating a responsibility*) facilitate subsequent movements of the hand in that direction (e.g., *away from self*; Glenberg et al., 2008b). The present study asks whether people might similarly use these types of experience-based representations to aid in conceptualizing abstract spatial concepts.

In contrast to abstract, intangible notions (e.g., *time, future*, *power*), people can directly perceive and act upon concrete space (e.g., *metric distance, a mountain*). As such, our interactions with the environment seem directly amenable to spatial representations that integrate perception and action. Indeed some recent work suggests that this is the case (Brunyé et al., 2010b; Wang et al., 2012). However, we also know that space can be thought about by using *abstract* concepts of world-centered reference systems, such as seen with canonical coordinate space (north, south, east, west). Even though people cannot directly perceive these directions, they can monitor them during navigation (Mark, 1989), and use them to shape mental representations of space (Tversky, 1993; Dabbs et al., 1998; Golledge, 1999). The coordinate terms *north, south, east* and *west* are completely abstracted from a body-referenced system and cannot be directly perceived in concrete space. In contrast, people can directly perceive *concrete* body-referenced directions along the *x, y*, and *z* planes; for instance, to the left or right along the mediolateral axis, and up or down relative to the dorsoventral axis. In spite of these differences, both abstract and concrete spatial concepts have been implicated in guiding and constraining spatial thought. Indeed, body-referenced directions are foundational to spatial language and communication, navigation, and the mental representation of large-scale space (Taylor and Tversky, 1996; Mou et al., 2004; Feist and Gentner, 2007). And even though people cannot directly perceive abstract coordinate directions, they can monitor them during navigation (Mark, 1989), and use them to shape mental representations of space (Tversky, 1993; Dabbs et al., 1998; Golledge, 1999).

The use of abstract spatial coordinate terms is critical for the transition from purely egocentric knowledge to the construction of allocentric representations (Hart and Moore, 1973). The ability to structure first-person experience using a fixed (i.e., self-abstracted) reference frame tends to develop between the ages of 5 and 11 (Herman and Siegel, 1978), and it is a difficult task, influenced by both spatial abilities and gender. For instance, those with generally high spatial abilities rely more upon coordinates during navigation; in contrast, those with lower spatial abilities rely more upon local landmarks and often find it difficult to use abstract spatial coordinate concepts when thinking about environments (for a review, see Wolbers and Hegarty, 2010). However, coding abstract coordinate directions in concrete space may provide a framework through which abstract spatial understanding can more easily manifest. In other words, binding self-relevant spatial references (e.g., *to the left*) to inherently self-abstracted coordinate directions (e.g., *west*) might facilitate transitions from egocentric to allocentric representational forms.

Along these lines, recent research has identified several peculiar tendencies when people attend to the north and south directions, which suggest that these coordinate directions are linked with some form of concrete space. For instance, people implicitly associate the north versus south with higher topography (Brunyé et al., in press) and social status (Gagnon et al., 2011). Further, there is consistent evidence, on both regional and international levels, that route planners tend to avoid routes that go initially northward versus southward, perhaps with the intention of avoiding more difficult locomotion (Brunyé et al., 2010a, 2012a). While the exact source of these effects remains unknown, it appears that people associate abstract concepts of coordinate space with concrete spatial axes. More specifically, people conceptualize north as up and south as down along the concrete vertical dimension (a concept first proposed, but not tested, by Shepard and Hurwitz, 1984). This association likely stems from the conventional orientation of north as up on maps; consistently viewing maps in this way may result in associating north with up, south as down, and east and west as right and left, respectively, relative to the self (see Brown and Levinson, 1993 for alternative hypotheses that might be applied to Tzeltal speakers). Grounding abstract concepts of coordinate space might allow people to transfer knowledge from experiential spatial domains in an effort to understand an otherwise intangible concept.

To test this possible association, we conducted two experiments using a masked priming paradigm designed to implicitly activate semantic concepts without conscious awareness, by measuring the effects of prime type (i.e., abstract coordinate terms) on dynamic motor responses to concrete target directions (i.e., concrete spatial terms or arrows). To track motor responses, we tracked mouse movements toward target directions using the freely availably Mouse Tracker software (Freeman and Ambady, 2010). Mouse Tracker records real time x and y mouse coordinates at an approximate 60–75 Hz sampling rate. Tracking mouse trajectories allows for examining the continuous temporal (i.e., when) and spatial (i.e., in which direction) dynamics of the comprehension process as it unfolds (Spivey et al., 2005; Dale et al., 2007). Tracking the kinematics of a response can be used to assess movement dynamics that hold potential for exposing cognitive operations that would otherwise be unexposed using traditional behavioral measures (cf., Abrams and Balota, 1991; Balota and Abrams, 1995; Spivey and Dale, 2004, 2006; Magnuson, 2005). Directly related to the present topic, work using mouse tracking demonstrates its utility in indexing the influence of metaphor processing on motor actions: when participants processed information regarding the past or future, their mouse movements were drawn toward the left or right, respectively (Miles et al., 2010a,b). Likewise this type of online measure may provide unique insights into the spatial and temporal nature of abstract-concrete spatial concept interactions.

The present work extends the current literature by examining three characteristics of the apparent link between abstract coordinate space and concrete vertical space. First, whereas prior research has identified an apparent link between north/south and up/down (Brunyé et al., 2010a, 2012a, in press), we propose that people may similarly represent east and west as to the right and left (respectively) with respect to the egocentric left-right axis. Second, we propose that these associations between abstract and concrete spatial concepts will alter movement trajectories when people are primed with abstract concepts and attempt to make hand movements that are either congruent or incongruent with the primed direction. Finally, we examine the temporal dynamics of any effects of abstract concept activation on motor movements. Our first experiment examined these issues by combining verbal primes (e.g., NORTH) with verbal targets (e.g., UP), and our second experiment tested whether our results would maintain with non-verbal targets (i.e., directional arrows) in an effort to rule out the possibility that lexical associations alone between primes and targets were driving our effects. Together, we provide the first demonstration that abstract spatial concept understanding is grounded in the perceptual motor system.

## Experiment 1

### Participants and design

One hundred Tufts University undergraduate students participated for monetary compensation. Informed consent was obtained from all participants in accordance with the Tufts University Institutional Review Board. All self-reported as right handed (using the Edinburg Handedness Inventory; Oldfield, 1971) and native English speaking. Given earlier work demonstrating that approximately 25–45% of participants report being prime-aware at similar prime durations (i.e., seeing a 43–45 ms prime; Bodner and Masson, 2003; Bodner and Dypvik, 2005), during debriefing we explicitly asked participants whether this was the case; 33 participants reported noticing at least one directional prime. Data from these prime-aware participants (*M*_age_ = 19.2; 13 male, 20 female) were removed, leaving 67 valid data sets for analysis (*M*_age_ = 19.9; 25 male, 42 female). Note that whereas shorter prime durations may reduce the number of prime-aware participants, they may not consistently achieve a semantic level of analysis (e.g., 25–40 ms; Holcomb et al., 2005; Klauer et al., 2007).

We used a masked priming procedure with a 4 (Prime Type: North, South, East, West, Center, Non-word) × 4 (Target Direction: Up, Down, Right, Left) within-participants design. Masked priming involves presenting words for such a brief duration that they activate cognitive processes without conscious awareness (Marcel, 1983; Cheesman and Merikle, 1984). In most cases, masks are used to flank (both prior to and after) the presentation of a prime with nonsense letter strings intended to minimize the chance that participants notice the presence of a prime or discriminate it from the nonsense letters (Dehaene et al., 1998; Lee et al., 1999). In the current study, we chose a masked priming manipulation to reduce the task demands characteristic of studies examining perceptuo-motor traces in memory; the greater the awareness of overlap between cueing and target stimuli, the more difficult it becomes to show strong evidence for spontaneous perceptuo-motor involvement in guiding human behavior (cf., Machery, 2007; Mahon and Caramazza, 2008; van Dantzig et al., 2008; Ditman et al., 2010).

We recorded mouse initiation times, response times, and movement trajectories over time using the freely available *Mouse Tracker* software (Freeman and Ambady, 2010).

### Materials

#### Primes and targets

We used six prime types corresponding to: the four coordinate prime directions (NORTH, SOUTH, EAST, WEST), one central control (CENTER), and a non-word control. A total of 36 non-word controls were generated using the ARC Non-word Database^1^, with each non-word ranging from 5 to 12 characters. A total of 100 forward and backward masks were generated using a Random Letter Sequence Generator^2^, with each sequence consisting of 12 letters (e.g., RVmoFcZNaDDu). Four target words were used (UP, DOWN, RIGHT, LEFT), referring to each of the four target locations.

#### Target array configuration

**Figure 1:**
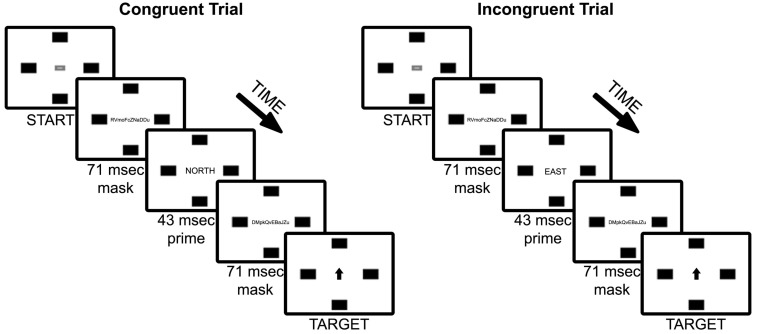
**Experiment 1 trial sequence of example congruent and incongruent (90°) trial types**. Note that font sizes of masks, primes, and targets are increased for figure legibility.

Using the Mouse Tracker software, we created an array of four black rectangular target boxes arranged along the horizontal and vertical axes on a 22′′ LCD monitor running at 1920 × 1200 resolution with a 70 Hz refresh rate (see Figure 1). At center, a START button was located in a gray rectangle. Each rectangular target was sized at approximately 15% of the corresponding monitor dimension (i.e., 290 w × 170 h pixels), and centered at a location 475 pixels from the START button. In standardized coordinate space (see *Data Scoring*), targets are centered at positions corresponding to −0.6 and 0.6 along the *x*-axis, and 0.15 and 1.35 along the *y*-axis.

### Procedure

Participants were instructed to “move the mouse as quickly as possible to the box that correctly corresponds with the word presented on the screen.” In a brief practice session, each participant was exposed to a series of 24 trials consisting of masked non-word primes, with six trials for each of the target rectangle locations (UP, DOWN, RIGHT, LEFT). At the beginning of each trial, the START button appeared at screen center. Upon left-clicking on the button with the mouse, the mouse cursor disappeared and the forward mask was presented for 71 ms, the prime for 43 ms, the backward mask for 71 ms, and then finally the target word (see Figure 1). The prime duration was selected based on work by Dehaene et al. (1998), which indicated that a 43 ms masked prime activated a semantic level of analysis as evidenced by both electrical brain activity (via event-related potentials) and hemodynamic response (via functional magnetic resonance imaging); specifically, Dehaene and colleagues found evidence that brain activation in response to masked word priming is not restricted to brain areas involved in sensory processing, but rather activated a range of brain mechanisms involved in perception, semantic categorization, and motor task preparation. Given these results, we expected that a 43 ms masked prime duration would be sufficient to activate semantic meaning of our coordinate primes (NORTH, SOUTH, EAST, WEST) without participants’ conscious awareness.

Once the target word was presented, the mouse cursor became visible and active and was centered on the START box; the participant then moved the mouse cursor to the target box and clicked the left mouse button. Participants always responded using their dominant (right) hand with a conventional two-button (plus scroll wheel) optical computer mouse positioned flat on the table ahead of and slightly to the right of the computer monitor. Following Freeman and Ambady (2010), participants were instructed if they either did not begin moving the mouse cursor within the first second it appeared after the target word was presented (“Please start moving earlier on, even if you are not fully certain of a response yet” was displayed at the end of the trial), if they did not respond within 4 s (“Over time!” was presented in red at the center of the screen and the trial was ended), or if an incorrect response was made an X appeared in red at the center of the screen.

Following practice, participants began the main experiment. Participants were presented with 204 trials consisting of 132 directionally primed trials (33 each of NORTH, SOUTH, EAST, WEST), 36 trials using the CENTER prime, and 36 trials using the control non-word prime. Each set of trials was divided amongst the four target directions (UP, DOWN, RIGHT, LEFT), and the 204 trials were presented in random order.

## Results

### Data scoring

The Mouse Tracker software samples x and y mouse cursor position every 13–16 ms from the point the mouse becomes active (target word presentation) to the response click. Incorrect trials are removed from further analysis, and all data undergo outlier trimming at 2.5 SD. Raw data from correct trials are rescaled to standardized coordinate space (*y*-axis range 0–1.5, *x*-axis range −1 to 1) and normalized over time using a linear interpolation process that results in 101 time steps (for more on this process, see: Spivey et al., 2005; Dale et al., 2007; Freeman et al., 2008, 2010; Freeman and Ambady, 2009, 2010). For each participant, we then averaged normalized data for each trial comprising each of our conditions.

Prior studies using mouse tracking have used several measures to quantify the differences in mouse trajectories relative to both optimal (vector-based) trajectories and across experimental conditions. These measures commonly include movement initiation time, movement duration, maximum deviation (MD), and area under the curve (AUC). Movement initiation time is the time (in ms) from the mouse becoming active to the participant beginning to move the mouse. Movement duration is the time (in ms) from the participant beginning mouse movement to clicking in the target region. MD is the peak amplitude of the movement trajectory relative to the optimal trajectory, and AUC is the area between the movement trajectory relative to the optimal trajectory. In general, MD and AUC are highly correlated and do not lead to different results (Freeman et al., 2008). In the present work, we analyze movement initiation time, movement duration, and to maintain compatibility with the extant literature we report MD (Freeman and Ambady, 2009; Miles et al., 2010a; Martens et al., 2012).

Thus, for each target direction (up, down, right, left) we plotted averaged and normalized mouse trajectories over time corresponding to each Prime Type.

### Analyses

**Figure 2:**
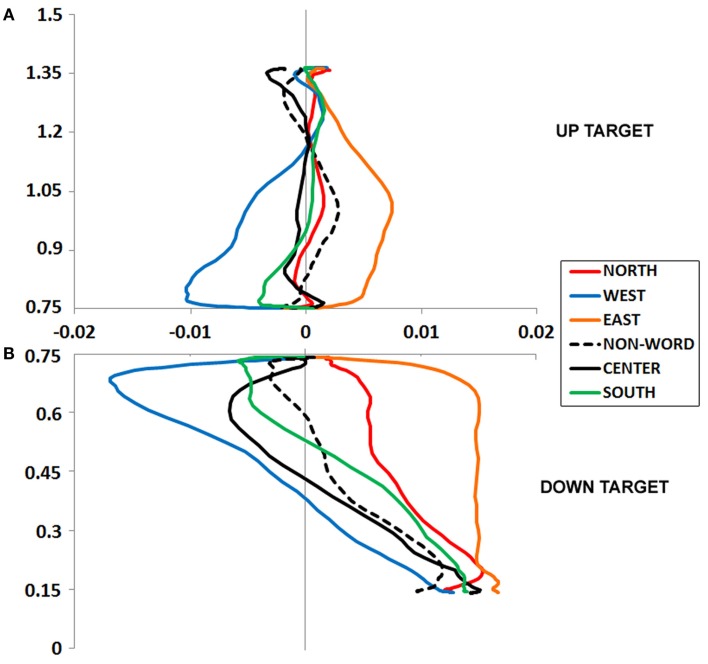
**(A,B)** Experiment 1 plotted movement trajectory data for the Up **(A)** and Down **(B)** target locations, for each of the six Prime Types.

**Figure 3:**
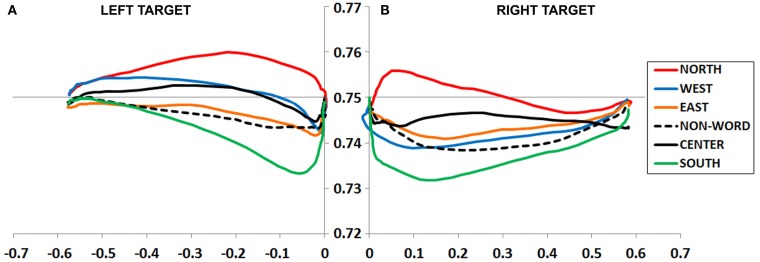
**(A,B)** Experiment 1 plotted movement trajectory data for the Left **(A)** and Right **(B)** target locations, for each of the six Prime Types.

We plotted data and conducted repeated-measures analyses of variance (ANOVAs) separately for each target direction (up, down, left, right). Movement duration data, along with statistical test results, are detailed in Table 1. Spatially and temporally normalized data are depicted in Figures 2 and 3 for each Prime Type. Finally, we provide MD data, along with statistical test results, also in Table 1. Note that follow-up analyses showed no main or interactive effects of participant gender (all *p*’*s* > 0.32).

**Table 1:** **Experiment 1 mean and standard error movement duration and maximum deviation (MD) data for each of the six prime types and four target types**.

Prime type	Target Type							
	Up		Down		Right		Left	
	*M*	SE	*M*	SE	*M*	SE	*M*	SE
**MOVEMENT DURATION**								
North	**704.3**	18.8	772.3**	16.6	695.1*	14.8	696.9	15.6
South	728.6^m^	19.1	**729.8**	18.3	695.9**	14.1	707.8*	15.3
East	742.9*	14.5	763.9^m^	16.0	**651.5**	17.4	679.3	14.8
West	747.1*	16.5	779.1**	15.1	681.7^m^	13.1	**669.9**	14.4
Non-word	727.1	16.4	757.4^m^	17.4	669.8	14.1	693.2^m^	14.4
Center	723.4	16.6	738.8	16.4	688.2*	14.2	687.6	13.9
**MAXIMUM DEVIATION (MD)**								
North	**0.0003**	0.005	0.016	0.008	0.007**	0.007	0.013*	0.004
South	−0.004	0.007	**0.002**	0.007	−0.022*	0.005	−0.017^m^	0.006
East	0.014**	0.004	0.022*	0.007	−**0.008**	0.005	−0.006	0.005
West	−0.011^m^	0.006	−0.012*	0.007	−0.011	0.004	−**0.003**	0.005
Non-word	0.0008	0.006	0.004	0.007	−0.011	0.005	−0.009	0.005
Center	−0.001	0.006	0.003	0.007	−0.009	0.005	0.0006	0.005

For each target type, we provide results from paired *t*-test comparing the congruent prime (e.g., north prime, up target; presented in **bold** print) versus each of the other 5 prime types (**p < 0.01, *p < 0.05, *^m^p* < 0.10).

### Up target

#### Up target mouse initiation time and movement duration

An ANOVA on mouse initiation times showed no effect of Prime Type (*F* < 1, *p* = 0.75; Figure 2A).

An ANOVA on movement duration times showed a marginal effect of Prime Type, *F*(5, 330) = 2.02, *p* = 0.07, η*^2^* = 0.03, with shortest movement durations following a North prime, and longest movement durations following an East or West prime. See Table 1 for results from paired tests comparing the North (congruent) to each of the five other primes.

#### Up target movement trajectory data

We calculated MD, or the peak amplitude of each movement trajectory relative to the optimal trajectory (vector from start to target); note that downward and leftward deviations are negative-going. These data were entered into an ANOVA, which revealed a marginal effect of Prime Type, *F*(5, 330) = 2.53, *p* < 0.05, η*^2^* = 0.04. As depicted in Figure 2A and detailed in Table 1, there was higher MD in the East prime condition relative to any other condition, and lower MD in the West prime condition relative to any other condition. In other words, the East prime biased Up target movement trajectories to the right, and West primes biased movement to the left.

### Down target

#### Down target mouse initiation time and movement duration

An ANOVA on mouse initiation times showed no effect of Prime Type (*F* < 1, *p* = 0.99; Figure 2B).

An ANOVA on movement duration times showed an effect of Prime Type, *F*(5, 330) = 3.39, *p* < 0.01, η*^2^* = 0.05, with shortest movement durations following a South prime, and longest movement durations following an East or West prime. See Table 1 for results from paired tests comparing the South (congruent) to each of the five other primes.

#### Down target movement trajectory data

An ANOVA on MD data revealed a main effect of Prime Type, *F*(5, 330) = 3.85, *p* < 0.01, η*^2^* = 0.05. As depicted in Figure 2B and detailed in Table 1, there was higher MD in the East prime condition relative to any other condition, and lower MD in the West prime condition relative to any other condition. In other words, as also seen in the Up target condition, the East prime biased movement trajectories to the right, and West biased them to the left.

### Left target

#### Left target mouse initiation time and movement duration

An ANOVA on mouse initiation times showed no effect of Prime Type (*F* < 1, *p* = 0.71; Figure 3A).

An ANOVA on movement duration times trended toward an effect of Prime Type, *F*(5, 330) = 1.5, *p* = 0.19, η*^2^* = 0.02, but did not reach significance. Numerically, there were shortest movement durations following a West prime, and longest movement durations following a North or South prime. See Table 1 for results from paired tests comparing the West (congruent) to each of the five other primes.

#### Left target movement trajectory data

An ANOVA on MD data revealed a main effect of Prime Type, *F*(5, 330) = 4.19, *p* < 0.01, η*^2^* = 0.06. As depicted in Figure 3A and detailed in Table 1, the highest MD occurred in the North prime condition, and lowest MD in the South prime condition. In other words, the North prime biased movement trajectories upward, and South biased them downward.

### Right target

#### Right target mouse initiation time and movement duration

An ANOVA on mouse initiation times showed no effect of Prime Type (*F* < 1, *p* = 0.82; Figure 3B).

An ANOVA on movement duration times showed an effect of Prime Type, *F*(5, 330) = 2.91, *p* < 0.05, η*^2^* = 0.04, with shortest movement durations following an East prime, and longest movement durations following a North or South prime. See Table 1 for results from paired tests comparing the East (congruent) to each of the five other primes.

#### Right target movement trajectory data

An ANOVA on MD data revealed a main effect of Prime Type, *F*(5, 330) = 3.66, *p* < 0.01, η*^2^* = 0.05. As depicted in Figure 3B and detailed in Table 1, the highest MD occurred in the North prime condition, and lowest MD in the South prime condition. In other words, the North prime biased movement trajectories upward, and South biased them downward.

### Experiment 1 discussion

Results demonstrate dynamic and directionally specific effects of abstract primes on movement trajectories toward concrete target directions. However, it is unclear whether these effects are restricted to conditions of using linguistic primes and targets; in other words, might low-level lexical associations between primes and targets (e.g., NORTH → → UP) be responsible for the present effects? We conducted a control experiment to test this possibility.

## Control Experiment

In this study, we replaced target words (UP, DOWN, LEFT, RIGHT) with arrows that pointed toward one of the four target directions. If our results are due to a metaphorical mapping between primed abstract coordinate directions and concrete target directions, and this effect exists above and beyond any simple lexical associations, then results should indicate similar movement trajectory biases to those found in Experiment 1.

### Participants and design

Fifty-nine Tufts University undergraduate students participated for monetary compensation, all right handed and native English speaking. Data from 9 prime-aware participants (*M*_age_ = 20.4; three male, six female) were removed from further analysis, leaving 50 valid data sets for analysis (*M*_age_ = 21.5; 15 male, 35 female).

The design matched that used in Experiment 1, with the 4 (Prime Type: North, South, East, West, Center, Non-word) × 4 (Target Direction: Up, Down, Right, Left) within-participants design.

### Materials and procedure

**Figure 4:**
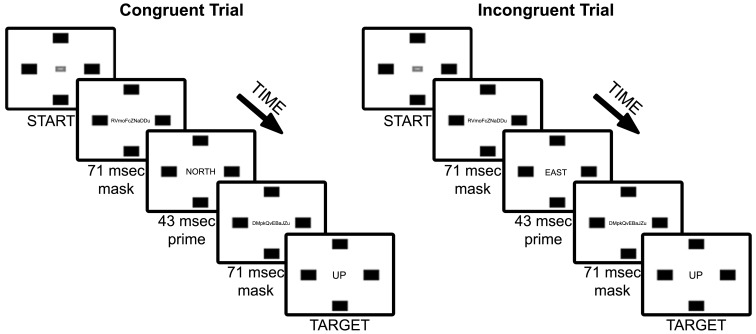
**Experiment 2 trial sequence of example congruent and incongruent (90°) trial types**. Note that font sizes of masks and primes, and drawn size of arrow, are increased for figure legibility.

All materials and procedures matched those of Experiment 1, with one exception: Rather than using target words, we used arrows that pointed in the direction of each of the four target locations (see Figure 4). Arrows were consistently sized (112 w × 57 h pixels) and rotated 90° to correspond to each of the four directions (UP, DOWN, LEFT, RIGHT).

## Results

### Data scoring and analyses

All data scoring and analyses matched those used in Experiment 1. As before, follow-up analyses showed no main or interactive effects of participant gender (all *p*’*s* > 0.27).

### Up target

#### Up target mouse initiation time and movement duration

**Figure 5:**
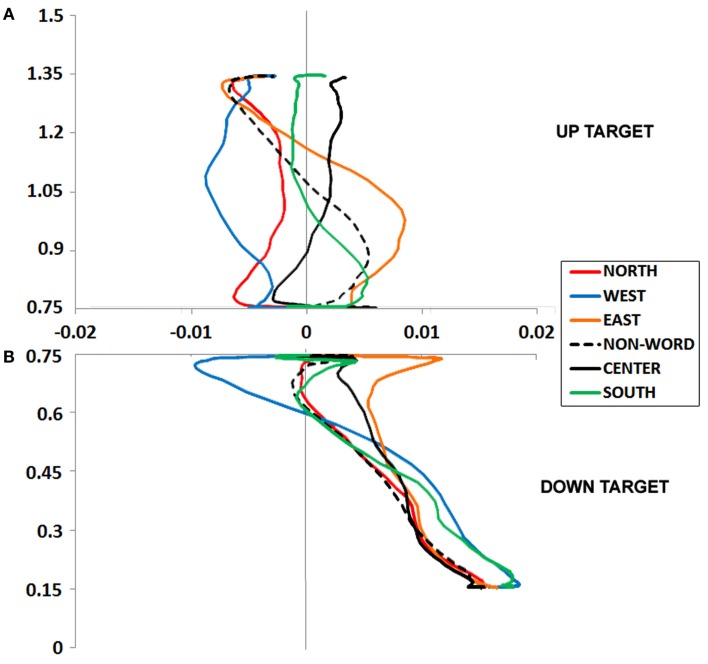
**(A,B)** Experiment 2 plotted movement trajectory data for the Up **(A)** and Down **(B)** target locations, for each of the six Prime Types.

An ANOVA on mouse initiation times showed no effect of Prime Type (*F* = 1.7, *p* = 0.13; Figure 5A).

An ANOVA on movement duration times showed a marginal effect of Prime Type, *F*(5, 245) = 1.96, *p* = 0.09, η*^2^* = 0.02, with shortest movement durations following a North prime, and longest movement durations following an East or West prime. See Table 2 for results from paired tests comparing the North (congruent) to each of the five other primes.

**Table 2:** **Experiment 2 mean and standard error movement duration and maximum deviation (MD) data for each of the six prime types and four target types**.

Prime type	Target type							
	Up		Down		Right		Left	
	*M*	SE	*M*	SE	*M*	SE	*M*	SE
**MOVEMENT DURATION**								
North	**1097.2**	14.9	1113.2	15.9	1047.3*	15.2	1078.2	21.4
South	1114.4	15.6	**1121.1**	18.9	1053.7^m^	16.7	1080.3^m^	18.1
East	1127.4*	14.4	1147.8	16.9	**1017.2**	17.8	1051.9	19.2
West	1133.3*	19	1150.8^m^	16.7	1010.6	15.4	**1057.8**	19.6
Non-word	1102.2	16.9	1128.1	16.3	1020.4	17.5	1048.5	16.9
center	1102.7	14.7	1118.2	17.9	1011.8	18	1037.4	16.7
**MAXIMUM DEVIATION (MD)**								
North	−**0.006**	0.006	0.013	0.008	−0.005	0.004	0.001*	0.005
South	0.001	0.008	**0.004**	0.009	−0.024**	0.004	−0.022^m^	0.004
East	0.01**	0.007	0.024*	0.024	−**0.016**	0.004	−0.006	0.006
West	−0.016	0.007	−0.009^m^	0.008	−0.011	0.004	−**0.012**	0.006
Non−word	−0.007	0.008	0.009	0.007	−0.016	0.005	−0.012	0.004
Center	0.003	0.006	0.016	0.007	−0.013	0.004	−0.005	0.005

Bold text indicates prime-target congruence. Superscript text indicates statistical significance: ^m^*p* < 0.10, *p < 0.05, **p < 0.01.

#### Up target movement trajectory data

Maximum deviation data were entered into an ANOVA, which revealed a main effect of Prime Type, *F*(5, 245) = 2.34, *p* < 0.05, η*^2^* = 0.05. As depicted in Figure 5A and detailed in Table 2, there was highest MD in the East prime condition and lowest MD in the West prime condition. In other words, the East prime biased Up target movement trajectories generally to the right, and West primes biased movement generally to the left.

### Down target

#### Down target mouse initiation time and movement duration

An ANOVA on mouse initiation times showed no effect of Prime Type (*F* < 1, *p* = 0.95; Figure 5B).

An ANOVA on movement duration times showed a marginal effect of Prime Type, *F*(5, 245) = 1.84, *p* = 0.10, η*^2^* = 0.04, with shortest movement durations following a South, North or Center prime, and longest movement durations following an East or West prime. See Table 2 for results from paired tests comparing the South (congruent) to each of the five other primes.

#### Down target movement trajectory data

An ANOVA on MD data revealed a main effect of Prime Type, *F*(5, 245) = 2.71, *p* < 0.05, η*^2^* = 0.05. As depicted in Figure 5B and detailed in Table 2, there was highest MD in the East prime condition and lowest MD in the West prime condition. In other words, as also seen in the Up target condition, the East prime biased movement trajectories generally to the right, and West biased them generally to the left.

### Left target

#### Left target mouse initiation time and movement duration

**Figure 6:**
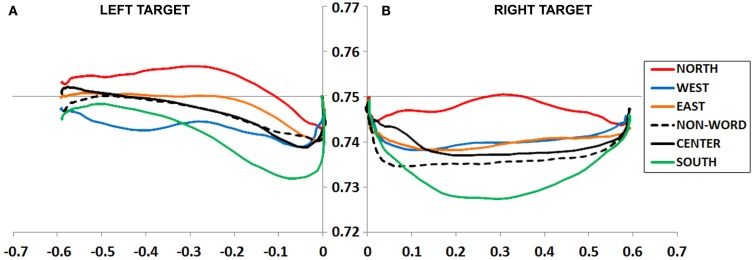
**(A,B)** Experiment 2 plotted movement trajectory data for the Left **(A)** and Right **(B)** target locations, for each of the six Prime Types.

An ANOVA on mouse initiation times showed no effect of Prime Type (*F* < 1, *p* = 0.83; Figure 6A).

An ANOVA on movement duration times revealed a marginal effect of Prime Type, *F*(5, 245) = 2.01, *p* = 0.08, η*^2^* = 0.04, with longest movement durations following a North or South prime. See Table 2 for results from paired tests comparing the West (congruent) to each of the five other primes.

#### Left target movement trajectory data

An ANOVA on MD data revealed a main effect of Prime Type, *F*(5, 245) = 2.75, *p* < 0.05, η*^2^* = 0.05. As depicted in Figure 6A and detailed in Table 2, the highest MD occurred in the North prime condition and lowest MD in the South prime condition. In other words, the North prime biased movement trajectories generally upward, and South biased them generally downward.

### Right target

#### Right target mouse initiation time and movement duration

An ANOVA on mouse initiation times showed no effect of Prime Type (*F* = 1.6, *p* = 0.16; Figure 6B).

An ANOVA on movement duration times showed an effect of Prime Type, *F*(5, 245) = 2.83, *p* < 0.05, η*^2^* = 0.05, with longest movement durations following a North or South prime. See Table 2 for results from paired tests comparing the East (congruent) to each of the five other primes.

#### Right target movement trajectory data

An ANOVA on MD data revealed a main effect of Prime Type, *F*(5, 245) = 3.01, *p* = 0.01, η*^2^* = 0.06. As depicted in Figure 6B and detailed in Table 2, the highest MD occurred in the North prime condition and lowest MD occurred in the South prime condition. In other words, the North prime biased movement trajectories generally upward, and South biased them generally downward.

## Discussion

When implicitly primed with abstract coordinate directions (north, south, east, west), participants revealed several biases toward the prime-congruent spatial directions when making seemingly simple mouse movements toward target locations. Specifically, mouse trajectories showed consistent and statistically robust movement biases that demonstrate an attraction toward primed abstract directions. When participants were tasked with making movements to the right or left, priming with the abstract spatial terms “north” or “south” biased trajectories upward and downward, respectively. Similarly, when moving up or down, priming with the abstract spatial terms “east” or “west” biased trajectories rightward and leftward, respectively. Experiment 2 demonstrated that these effects exist above and beyond any influence of relatively low-level lexical associations between primed and target words. Together, these results provide the first evidence that people ground abstract spatial concepts in perceptuo-motor systems. This grounding mechanism is evidenced through biased movement trajectories that occur even when abstract concepts are activated outside of participants’ awareness.

These results are consistent with research demonstrating that abstract concepts are frequently understood through metaphorical mappings to concrete experienced space (Barsalou, 1999; Boroditsky and Prinz, 2008; Miles et al., 2010a; Gagnon et al., 2011). Presently, it seems that people associate abstract spatial concepts with concrete spatial experience, and these associations are grounded in body-referenced axes. These body-referenced axes extend left-right along the mediolateral axis, and up-down along the vertical axis.

The associations between abstract and concrete space likely arise through simple correlational learning (Hebb, 1949); daily experiences viewing maps, atlases, and even navigation devices consistently associate north with the upward direction (and south with down). These experiences give rise to associations between east and right, and west and left that seem to exist even outside of participants’ awareness. Indeed, free-association norms (Nelson et al., 1998) show very weak associations between abstract and concrete spatial terms, and implicit (but not explicit) associations between north and upward space predict route planning biases toward the south (Brunyé et al., in press). We also note, however, that mouse tracking is limited with regard to determining whether north and south are represented along the forward-backward (anteroposterior) versus up/down body axes; moving the mouse forward/backward results in translational movement on the computer monitor along the up/down axis. Thus, there is some conflict between the motoric and perceptual representations of mouse movement along the *y*-axis. Though we have not tested between these axes, we expect that grounding abstract spatial concepts occurs along both forward/backward and up/down body-referenced axes; indeed some early work suggests that people may associate north with the forward egocentric direction (Shepard and Hurwitz, 1984), and our own work suggests associations between north and vertical perceived space (e.g., spatial topography; Gagnon et al., 2011).

The ability to track the online dynamics of mouse movement trajectories shows promise in revealing otherwise hidden cognitive operations (Spivey and Dale, 2006; Miles et al., 2010a). In the current study, response time patterns did not reveal consistent evidence for priming effects; for example, response times for moving the mouse to the left were not significantly affected by any prime type. Only when examining movement dynamics were we able to identify consistent evidence that abstract spatial concepts may be grounded in concrete representations of body-referenced space. Thus, relying exclusively on more traditional response measures does not always reveal the true dynamics of information processing as it unfolds over time. In this manner, movement trajectories are proving valuable in examining the spatial component of mental activity (Oliveri et al., 2009). The present data suggest a rather specific time course for abstract concept activations influencing movement trajectories.

In addition, mouse initiation times were not affected by spatial primes, suggesting relatively delayed onset of abstract concept priming effects on movement trajectories. It could be the case that motor movement trajectories were altered through a cascading activation mechanism that activates abstract conceptual content which then, in turn, influences the comprehension of subsequent content (Mahon and Caramazza, 2008). This type of effect would be congruent with work demonstrating that the motor system is activated approximately 200 ms following the presentation of a body-relevant action word. In the present design, if primed concepts are only beginning to activate the motor system by the time our target words are presented (71 ms after the prime), behavior becomes biased by the prime only after the onset of target-directed movement.

The present results speak to strong relationships between the ways in which we perceive and interact with our environment on a daily basis and the ways in which we process and represent abstract information. The idea that abstract concepts are bound to experience is foundational to theories of embodied cognition (Lakoff and Johnson, 1999; Barsalou, 2008), which posit that mental representations of both concrete and abstract concepts often reflect specific regularities in the way we perceive and interact with those concepts in the world (cf., Miles et al., 2010a). Through this theorized mechanism, people process abstract, intangible concepts through real-world perceptions and actions. In many cases this grounding mechanism facilitates deeper understanding of the abstract (Barsalou, 1999). In the context of this experiment, coding abstract coordinate directions in concrete space may provide a framework through which abstract spatial understanding can more easily manifest. More specifically, thinking about coordinate directions (e.g., *west*) in terms of self-relevant space (e.g., *to the left*), may facilitate the construction of allocentric mental models from egocentric experience. Interestingly, some languages such as Guugu Yimithirr (northeastern Australia) encode directions using world-centered reference systems; for instance, referring to sides of the body as east and west dependent on the facing direction of the individual (e.g., the bug is on your west arm; Brown and Levinson, 1992, 1993; Haviland, 1996, 1998). To our knowledge, no work has considered whether these individuals show any preferred association between these directions and sides of their body, or if they show relatively facilitated generation of allocentric models.

Several theoretical positions have been offered to account for the types of results described presently. Conceptual metaphor theory would suggest that image schemas of directly experienced axes (up, down, left, right) are necessary to structure abstract spatial concepts in order to facilitate understanding (Lakoff and Johnson, 1980; Gibbs, 2003); under this theory, people form image schemas to represent spatial relations, and this metaphorical mapping is reflected in language (e.g., *our relationship is headed south*.). A second position posits that understanding abstract language recruits the motor system (Glenberg et al., 2008a,b); under this theory, both concrete and abstract language that describe or imply (respectively) directional motion are at least partially understood through activation of the motor system. Perhaps the most extreme position posits that perceptuo-motor representations are not only involved but also necessary elements underlying the ability for humans to understand abstract concepts (Barsalou, 1999; Barsalou and Wiemer-Hastings, 2005). Somewhat more even-handed treatments suggest that both linguistic and perceptuo-motor representations influence the comprehension of abstract concepts; for instance, while abstract thought might not necessitate perceptual or motoric simulation, these processes might serve to enrich linguistic representations and facilitate deeper understanding (Mahon and Caramazza, 2008; Dove, 2009; Pecher et al., 2011).

We propose, congruent with some earlier claims regarding metaphor processing (Murphy, 1996; Pecher et al., 2011), that the abstract concept of coordinate direction likely has a learned structure of its own, as do body-referenced concrete directions. In other words, abstract coordinate direction *can* be understood without association to concrete space, though this concrete association is frequently relied upon to structure understanding. This may be particularly the case with individuals who find it difficult to understand abstract spatial concepts, such as those with lower spatial ability; future work might examine how spatial abilities modulate propensities toward mapping abstract spatial concepts to concrete space. Regardless of their precise source, however, even though abstract-concrete associations (i.e., north-up) might aid understanding in many situations, we propose that they might also impair certain types of behavior. For instance, our recent work suggests that implicit associations between abstract coordinates and concrete vertical space lead people to misassociate the northward direction with upward movement and thus greater physical exertion (due to gravity; Brunyé et al., in press); because action planning and perception involve assessments of predicted body states and affordances, the associations revealed in the present work might bias route planning and navigation decisions in unintended manners, potentially leading to suboptimal spatial decisions (Knoblich and Flach, 2001; Witt et al., 2004; Fajen, 2005; Proffitt, 2006).

We introduced the present research as having three main goals and hypotheses. First, we expected that people would associate abstract coordinate terms (i.e., cardinal directions) with egocentric body axes (up/down, left/right); response time data showed some support for this claim, with slower response times when participants were primed with a direction orthogonal to their target movement direction. Second, we expected that movement trajectories would prove valuable in elucidating otherwise hidden cognitive operations; movement trajectory data showed strong support for this claim, with reliable directionally specific movement biases toward orthogonal primes. Finally, we set out to examine the temporal dynamics of abstract concept activation influences on motor movements; results showed strong evidence for a temporally defined process by which abstract concepts alter movement trajectories. Together, we provide the first demonstration that abstract spatial concept understanding is bound to concrete space and can manifest through the perceptuo-motor system.

## Conflict of Interest Statement

The authors declare that the research was conducted in the absence of any commercial or financial relationships that could be construed as a potential conflict of interest.

## Footnotes

^1^http://www.maccs.mq.edu.au/~nwdb/nwdb.html

^2^http://www.dave-reed.com/Nifty/randSeq.html
